# Menaquinone-4 Amplified Glucose-Stimulated Insulin Secretion in Isolated Mouse Pancreatic Islets and INS-1 Rat Insulinoma Cells

**DOI:** 10.3390/ijms20081995

**Published:** 2019-04-23

**Authors:** Hsin-Jung Ho, Hitoshi Shirakawa, Keisukei Hirahara, Hideyuki Sone, Shin Kamiyama, Michio Komai

**Affiliations:** 1 Laboratory of Nutrition, Graduate School of Agricultural Science, Tohoku University, Sendai 980-8572, Japan; kinyou@g-mail.tohoku-university.jp (H.-J.H.); keihira7@gmail.com (K.H.); mkomai@m.tohoku.ac.jp (M.K.); 2 International Education and Research Center for Food Agricultural Immunology, Graduate School of Agricultural Science, Tohoku University, Sendai 980-8572, Japan; 3 Department of Health and Nutrition, Faculty of Human Life Studies, University of Niigata Prefecture, Niigata 950-8680, Japan; sone@unii.ac.jp (H.S.); kammy@unii.ac.jp (S.K.)

**Keywords:** menaquinone-4, cAMP/Epac pathway, glucose-stimulated insulin secretion

## Abstract

Vitamin K2 is indispensable for blood coagulation and bone metabolism. Menaquinone-4 (MK-4) is the predominant homolog of vitamin K2, which is present in large amounts in the pancreas, although its function is unclear. Meanwhile, β-cell dysfunction following insulin secretion has been found to decrease in patients with type 2 diabetes mellitus. To elucidate the physiological function of MK-4 in pancreatic β-cells, we studied the effects of MK-4 treatment on isolated mouse pancreatic islets and rat INS-1 cells. Glucose-stimulated insulin secretion significantly increased in isolated islets and INS-1 cells treated with MK-4. It was further clarified that MK-4 enhanced cAMP levels, accompanied by the regulation of the exchange protein directly activated by the cAMP 2 (Epac2)-dependent pathway but not the protein kinase A (PKA)-dependent pathway. A novel function of MK-4 on glucose-stimulated insulin secretion was found, suggesting that MK-4 might act as a potent amplifier of the incretin effect. This study therefore presents a novel potential therapeutic approach for impaired insulinotropic effects.

## 1. Introduction

Vitamin K (VK) is a fat-soluble vitamin that functions as a cofactor for microsomal γ-glutamyl carboxylase and has a distinct role in the posttranslational carboxylation of glutamate to γ-carboxyglutamate (Gla) residues of VK-dependent proteins. VK exists in two natural forms, VK1 (phylloquinone, highly abundant in leafy greens) and VK2 (menaquinone, contained in dairy products and fermented foods). VK1 is the major form of dietary VK; however, VK2, especially menaquinone-4 (MK-4), is the major form in animal tissues. MK-4 is a homolog of VK2 and is formed by the conversion of a part of ingested VK1 [1]. Certain functions of MK-4, besides its well-known roles in blood coagulation and bone metabolism via Gla modification, have been confirmed, including the induction of apoptosis in tumor cells [2,3,4,5], modulation of the nuclear receptor SXR/PXR [6,7,8], anti-inflammatory activity in lipopolysaccharide-induced models [9,10], and the enhancement of testosterone production [11]. Recent studies have also revealed that MK-4 can be used for the treatment of osteoporosis [12,13,14,15]. In an earlier study, we showed that MK-4 is widely present in the body, especially in the pancreas [16]; however, the role of MK-4 in the pancreas remains uncertain.

Glucose-stimulated insulin secretion (GSIS) is a complicated metabolic mechanism for maintaining glucose homeostasis in pancreatic islet β-cells. As shown in Supplementary Figure S1, the presence of β-cells with a stimulatory concentration of glucose elicits rapid insulin release by elevating intracellular ATP concentrations, accompanied by the closure of the ATP-sensitive K^+^ (KATP) channels in the plasma membrane. Membrane depolarization occurs subsequent to open voltage-dependent Ca^2+^ channels, which mediates insulin granule exocytosis [17,18]. Meanwhile, gut-derived incretins such as glucose-dependent insulinotropic peptide (GIP) and glucagon-like peptide-1 (GLP-1) play a major role in the postprandial regulation of insulin secretion, accounting for at least 50% of total insulin. To lower blood glucose levels, incretins are secreted from enteroendocrine cells to activate cAMP signaling for amplifying nutrient-induced insulin secretion [19,20,21] and insulinotropic effects, including the inhibition of glucagon secretion [22] and decreased endogenous glucose production [23]. It is well established that type 2 diabetes mellitus (T2DM) generally results from the progressive failure of β-cell function, followed by a reduction in insulin secretion, which may actually aggravate the disease [24,25]. Insulin secretagogues have been widely used for managing T2DM; however, because the drug triggers insulin secretion irrespective of glucose concentrations, iatrogenic hypoglycemia remains an impediment [26,27].

Recently, VK has been shown to improve insulin sensitivity and glycemic status and to reduce the risk of T2DM in several clinical cases [28,29,30,31,32,33]. Sakamoto et al. found that low dietary VK intake appeared to induce poor early insulin response after glucose loading [34,35]. These results prompt us to surmise that VK plays a beneficial role in insulin sensitivity and glucose metabolism, decreasing the risk of cardiovascular disease, T2DM, and metabolic syndrome. However, the regulation of insulin by VK, especially MK-4, has not been well elucidated. The aim of the present study is to clarify the role of MK-4 on GSIS in β-cells.

## 2. Results

### 2.1. MK-4 Enhanced GSIS in Isolated Mouse Pancreatic Islets and Rat INS-1 Cells

**Figure 1:**
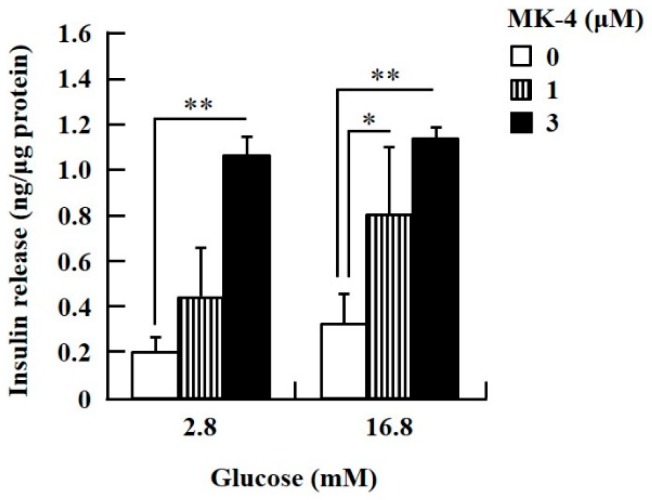
MK-4 regulated GSIS in INS-1 cells. Cells were treated with the indicated concentrations of glucose and MK-4 for 1 h. Data are presented as mean ± SD (*n* = 3); *p < 0.05, **p < 0.01.

**Figure 2:**
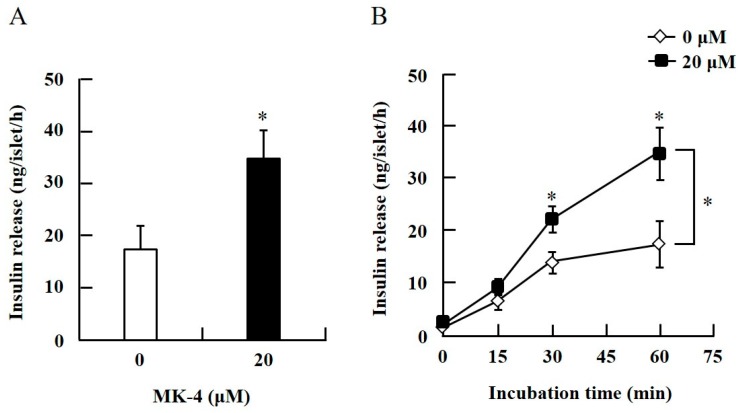
MK-4 regulated GSIS in mouse pancreatic islets. Isolated islets were treated with 20 µM MK-4 (**A**) or incubated time (**B**) with 2.8 mM Glc for 1 h. Data are presented as mean ± SE (*n* = 5); *p < 0.05.

To confirm the effect of MK-4 on GSIS, isolated islets and INS-1 cells were used in the present study. To determine the dosage of MK-4 for INS-1 cells, we analyzed the cell survival rate under 0–100 µM MK-4 treatment for 24 h by using the WST-1 assay (Supplementary Figure S2), which suggested that the concentration of 3 µM MK-4 was harmless to the cells. Accordingly, MK-4 at 1 and 3 µM was used in INS-1 cells for the subsequent experiments. We then evaluated the effect of MK-4 on GSIS in INS-1 cells, as shown in Figure 1. MK-4 enhanced insulin levels in both basal (2.8 mM) and high (16.8 mM) glucose-stimulated INS-1 cells in a dose-dependent manner. We used mouse pancreatic islets isolated from genetically diabetic KK-Ay mice subsequently. The isolated pancreatic islets were stimulated with 2.8 mM Glc and MK-4 for 1 h, and 20 µM MK-4 was found to enhance GSIS about two fold compared with the 0 µM MK-4 group; thus, MK-4 amplified GSIS in the isolated mouse pancreatic islets of KK-Ay mice (Figure 2). Moreover, we also found that MK-4 enhanced GSIS in C57BL/6J mice (Supplementary Figure S3). Hence, MK-4 enhanced insulin secretion in both isolated islets and INS-1 cells under glucose stimulation, and pancreatic β-cells were the target of MK-4.

### 2.2. MK-4 Increased cAMP Levels in INS-1 Cells

**Figure 3:**
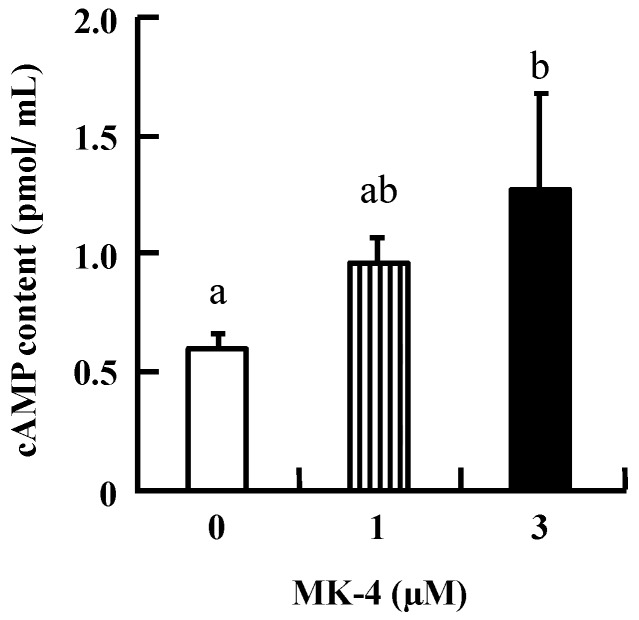
MK-4 stimulated intracellular cAMP levels in INS-1 cells. Cells were treated with the indicated concentrations of MK-4 for 1 h. Data are presented as mean ± SD (*n* = 3). Different letters indicate significant differences (*p* < 0.05).

To investigate the mechanism by which MK-4 regulates GSIS in β-cells, INS-1 cells were used in the following experiments. Initially, intracellular cAMP was measured in INS-1 cells because increased cAMP levels promote nutrient-induced insulin secretion in enteroendocrine cells. As shown in Figure 3, the level of cAMP tended to increase with the lower dose (1 µM MK-4), and was significantly increased in the group treated with 3 µM MK-4 for 1 h. These results revealed that MK-4 might enhance insulin secretion by regulating the cAMP-dependent pathway in INS-1 cells.

### 2.3. MK-4 Amplified GSIS by Regulating the cAMP/Epac-Dependent Pathway but not the cAMP/PKA-Dependent Pathway in INS-1 Cells

**Figure 4:**
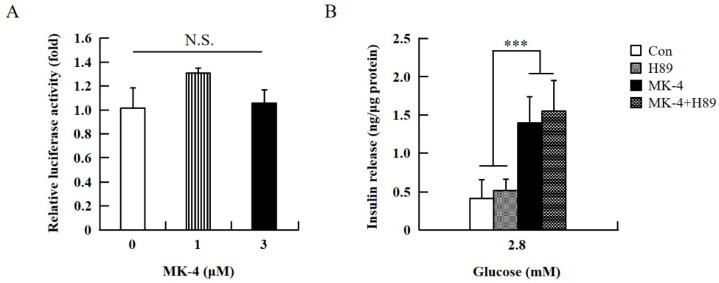
Effect of MK-4 on the activation of PKA in INS-1 cells. (**A**) Effects of MK-4 on luciferase activity in INS-1 cells. Cells were transfected with a CRE-inducible reporter gene and then treated with MK-4 for 3 h. (**B**) Effects of a PKA inhibitor (H89) on GSIS in INS-1 cells. Cells were treated with the indicated concentrations of glucose, MK-4 (3 µM), and H89 (10 µM) for 1 h. Insulin concentrations were measured by EIA. Data are presented as mean ± SD (*n* = 3); ***p < 0.001.

**Figure 5:**
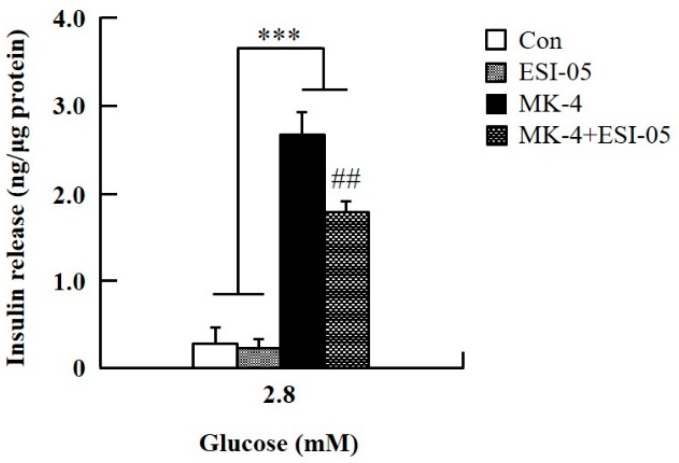
Effect of MK-4 on the activation of Epac in INS-1 cells. Cells were treated with the indicated concentrations of glucose, MK-4 (3 µM), and Epac2 inhibitor (ESI-05; 10 µM) for 1 h. Data are presented as mean ± SD (*n* = 3); ***p < 0.001 vs. MK-4/MK-4+ESI-05, ##*p* < 0.001 vs. MK-4.

To verify the effect of MK-4 on cAMP-dependent pathways, the cAMP/PKA pathway was investigated first (Figure 4). However, through the cAMP response element (CRE)-reporter gene assay, it was found that luciferase activity did not change with either 1 or 3 µM of MK-4 treatment (Figure 4A). Not unexpectedly, treatment with the protein kinase A (PKA) inhibitor H89 also did not affect the influence of MK-4 on GSIS (Figure 4B), which showed that MK-4 might not regulate PKA activity in INS-1 cells. Therefore, we analyzed another cAMP-dependent pathway, the cAMP/Epac2 pathway of GSIS, in INS-1 cells. We blocked the cAMP/Epac2 pathway using the exchange protein directly activated by the cAMP 2 (Epac2) inhibitor ESI-05. As shown in Figure 5, the insulinotropic effect of MK-4 might have been abolished by the Epac2 inhibitor, suggesting that MK-4 amplified GSIS through the regulation of the cAMP/Epac-dependent pathway but not the cAMP/PKA-dependent pathway in INS-1 cells.

## 3. Discussion

Incretins modulate insulin signaling to regulate energy balance and glucose homeostasis between the fasting state and the fed state. Evidence suggests that the incretin effect is blunted in T2DM patients, most likely as a consequence of the diabetic state [36,37,38,39], and the disruption of the insulinotropic effect of incretins particularly occurs in glucose homeostatic dysregulation. Thus, there is no reasonable doubt that the attenuation of the incretin effect contributes to the glucose intolerance of T2DM patients.

Recent studies have highlighted the possibility of the therapeutic applications of incretins, including two types of agents: incretin mimetics and incretin effect amplifiers [40,41]. GLP-1 mimetics increase plasma GLP-1 concentration, thereby contributing to the decrease in HbA1c, fasting blood glucose, and body weight [42]. Although treatment with GLP-1 mimetics has been claimed to cause acute pancreatitis or renal impairment in humans [43,44,45,46], no causal relationship with the mimetics has been shown, and no evidence has been observed in mice, rats, or monkeys injected with a GLP-1 mimetic (liraglutide) at a dose 60 times that used for humans [47]. On the other hand, dipeptidyl peptidase-4 (DPP-4) inhibitors as incretin amplifiers suppress plasma glucagon and reduce blood levels of HbA1c and glucose without changes in body weight [48]. One study indicated that DDP-4 inhibitors did not confer an increased risk of pancreatitis in T2DM patients [49]; however, some common side effects, such as headache, nasopharyngitis, upper respiratory infections, urinary system infections [50], severe allergic reactions [51], and hypoglycemia [52], have been reported. A previous study mentioned that GSIS was impaired in islets from KK-Ay mice, an animal model of T2DM [53], suggesting it is a suitable model for investigating the metabolic mechanism of GSIS. Our preliminary experiment showed that KK-Ay mice fed a VK-deficient diet had lower plasma insulin levels after glucose loading compared to mice fed a control diet [Hirahara et al., unpublished data]. Moreover, dietary VK levels and blood insulin levels have been found to be positively correlated with blood insulin levels after glucose loading in rats and humans [34,35]. We first reported that MK-4 is a potent stimulator (incretin amplifier effect) of GSIS in β-cells of the T2DM animal model, KK-Ay mice.

It is known that cAMP regulates exocytosis in various secretory cells, including β-cells, where cAMP plays a role as the most important potentiator for insulin secretion in a glucose concentration-dependent manner. The amplification of cAMP in insulin secretion via both a PKA-dependent and a PKA-independent manner reveal a novel Epac (also known as cAMP-regulated guanine nucleotide exchange factors; cAMPGEFs)-dependent pathway. Although the effect of cAMP on the exocytosis of insulin is generally thought to be through the activation of PKA, several studies have indicated that the PKA-independent mechanism involving the cAMP/Epac2 pathway is crucial in the potentiation of insulin secretion by incretins [54,55,56,57]. cAMP has also been proposed to mediate exocytosis directly in a PKA-independent manner in pancreatic islet β-cells. Among current antidiabetic drugs, sulfonylureas have been extensively used for nearly 50 years, and Epac2 is a direct target of sulfonylurea [58]. cAMP-potentiated fusion events of glucose-induced exocytosis are markedly reduced in Epac2-deficient mice [57], indicating that Epac2 is essential in GSIS potentiated by cAMP. These findings together support Epac2 as a target for diabetes therapy. Here, we found that MK-4 might function as an incretin-like nutrient that amplifies GSIS via elevated cAMP levels, followed by Epac2 regulation, but does not enhance the activation of PKA although cAMP can stimulate both Epac2 and PKA. We previously showed that MK-4 stimulates the cAMP/PKA signaling pathway in testis-derived cells and then enhances testosterone production [11,59]. However, the dissimilar effects of MK-4 on the cAMP dependent pathway has not been elucidated. Further studies are needed to fully understand the complex and multifactorial mechanisms of MK-4 involved in GSIS.

## 4. Materials and Methods

### 4.1. Reagents

MK-4 was obtained from Nisshin Pharma Inc. (Tokyo, Japan) and dissolved in ethanol to obtain a stock solution (50 mM); it was then stored in the dark at −20 °C. H89 (Sigma-Aldrich, St. Louis, MO, USA) and ESI-05 (4-methylphenyl-2,4,6-trimethlyphenylsulfone; BIOLOG Life Science Institute, Bremen, Germany), which are inhibitors of PKA and Epac2, respectively, were dissolved in dimethyl sulfoxide (Sigma-Aldrich) to obtain stock solutions (10 mM) at −20 °C.

### 4.2. Animals and Isolation of Pancreatic Islets

Four-week-old male KK-Ay mice were purchased from CLEA Japan Inc. (Tokyo, Japan) and were housed at 23 °C ± 1 °C, 60% ± 5% humidity, and a 12 h:12 h light/dark cycle. The experiments were approved by the Animal Care Committee of Tohoku University (2013AgA-023, 2016AgA-023; approved at 13 February 2013 and 24 March 2016, respectively).

For the isolation of pancreatic islets from mice, collagenase (Type V, Sigma-Aldrich) with Krebs–Ringer bicarbonate HEPES (KRBH) buffer [1.29 mM NaCl, 5 mM NaHCO_3_, 4.7 mM KCl, 1.2 mM KH_2_PO_4_, 1.2 mM MgSO_4_, 10 mM HEPES (pH 7.4), and 0.1% bovine serum albumin (BSA)] was injected into the pancreas via the common bile duct as described previously [60], followed by enzymatic digestion of the pancreas in the collagenase solution at 37 °C for 15 min in a flask. Next, mechanical digestion was proceeded by gentle pipetting to separate islets from pancreatic tissue. The fragmented pancreatic tissue was spun down at 800 rpm for 30 s, and the supernatant was discarded. The fragmented pancreatic tissue was gently resuspended with 5 mL KRBH buffer, after which the solution was moved into a culture dish containing KRBH buffer on ice, and the islets were handpicked into another dish for further experiments. The isolated islets were used within 2 h in this study.

### 4.3. Glucose-Stimulated Insulin Secretion in Isolated Islets

For the stabilization of the isolated islets, 5 islets/tube were pre-incubated in KRBH buffer with 2.8 mM glucose (Glc) at 37 °C for 1 h. The supernatants were discarded, and the islets were refreshed with KRBH buffer with 2.8 mM Glc and 20 µM MK-4 at 37 °C for 0, 15, 30, and 60 min. The collected KRBH buffer was centrifuged at 1000× *g* for 5 min, and the supernatants were stored at −20 °C. Insulin concentrations were measured using a mouse insulin EIA kit (Shibayagi, Gunma, Japan) following the manufacturer’s instructions.

### 4.4. Cell line and Culture Conditions

The INS-1 rat insulinoma cell line was a kind gift from Dr. Harada, Osaka Prefecture University [61,62,63]. INS-1 cells were maintained in RPMI-1640 medium (Sigma-Aldrich) supplemented with 11.1 mM glucose, 30 mM NaHCO_3_, 1 mM sodium pyruvate, 10 mM HEPES, 5 µL 2-mercaptoethanol, 10% fetal bovine serum (Biosera, Boussens, France), 50 U/mL penicillin, and 50 µg/mL streptomycin in a 5% CO_2_ humidified incubator at 37 °C [64]. For experiments, INS-1 cells were used between passages 21 and 30. The INS-1 cells were passaged and used at 60–70% confluence.

### 4.5. Cell Proliferation Assay

INS-1 cells were seeded into 96-well plates at a density of 1.0 × 10^4^ cells/well and incubated overnight. The medium was replaced the following day with 0–100 µM MK-4. Following incubation for 24 h, the number of viable cells in each sample was determined using the Premix WST-1 Cell Proliferation Assay System according to the manufacturer’s instructions (Takara Bio Inc., Kusatsu, Japan).

### 4.6. GSIS in INS-1 Cells

INS-1 cells were seeded into 24-well plates at a density of 0.5 × 10^4^ cells/well and incubated overnight. The medium was removed the following day, and HEPES buffer [KRBH for cells, 135 mM NaCl, 3.6 mM KCl, 0.5 mM NaH_2_PO_4_, 0.5 mM MgCl_2_, 1.5 mM CaCl_2_, 5 mM NaHCO_3_, 10 mM HEPES (pH 7.4), and 0.2 % BSA] with 2.8 mM glucose was added into wells and pre-incubated for 1 h. Next, HEPES buffer was replaced with 2.8 and 16.8 mM glucose containing 0–3 µM MK-4 for 1 h. The collected HEPES buffer was centrifuged at 1000× *g* for 5 min, and the supernatant was stored at −20 °C. Insulin concentrations were determined with a rat insulin EIA kit (Morinaga, Tokyo, Japan) following the manufacturer’s instructions. Protein concentrations were measured by the Lowry method to normalize the insulin concentrations. For PKA and Epac2 inhibition experiments, H89 and ESI-05 were used as PKA and Epac2 inhibitors, respectively. Cells were pre-incubated in HEPES buffer with 2.8 mM glucose and then replaced with fresh HEPES buffer containing MK-4 and pathway inhibitors for 1 h. Insulin concentration was measured in the collected HEPES buffer.

### 4.7. Measurement of cAMP Levels

INS-1 cells were seeded in 60 mm dishes and incubated overnight. The culture medium was refreshed with MK-4-containing medium, followed by incubation for 1 h. cAMP concentrations in cell lysates were determined with a cAMP EIA kit according to the manufacturer’s instructions (Cayman Chemical, Ann Arbor, MI, USA). The detailed procedure of the luciferase reporter assay is available in a previous publication [64].

### 4.8. CRE-Reporter Gene Assay

INS-1 cells were transiently transfected by using the FuGENE HD transfection reagent (Promega, Madison, WI, USA) with the pGL4.29 (Promega) plasmid harboring the firefly luciferase gene under the control of the CRE; the pCH110 plasmid containing the β-galactosidase reporter gene [65] was used as an internal control. Cells were stimulated by MK-4 for 3 h. The luciferase reporter assay was carried out as described in an earlier study [64].

### 4.9. Statistical Analysis

Data are expressed as mean ± SD and were evaluated by one-way or two-way analysis of variance, followed by Tukey’s honestly significant difference test using SAS v.9.3 software (SAS Institute, Cary, NC, USA); *p*-values of <0.05 were considered significant.

## Supplementary Materials

Supplementary materials can be found at https://www.mdpi.com/1422-0067/20/8/1995/s1.

## Abbreviations

MK-4	menaquinone-4
GSIS	glucose-stimulated insulin secretion
GIP	glucose-dependent insulinotropic peptide
GLP-1	glucagon-like peptide-1
T2DM	type 2 diabetes mellitus
CRE	cAMP response element
DDP-4	dipeptidyl peptidase-4
PKA	protein kinase A
Epac2	exchange protein directly activated by cAMP 2
