# Supplementary figures and images for: Menaquinone-4 Amplified Glucose-Stimulated Insulin Secretion in Isolated Mouse Pancreatic Islets and INS-1 Rat Insulinoma Cells
Source: Int J Mol Sci. 2019 Apr 23;20(8):1995. doi: 10.3390/ijms20081995 (PMC6515216; doi:10.3390/ijms20081995)

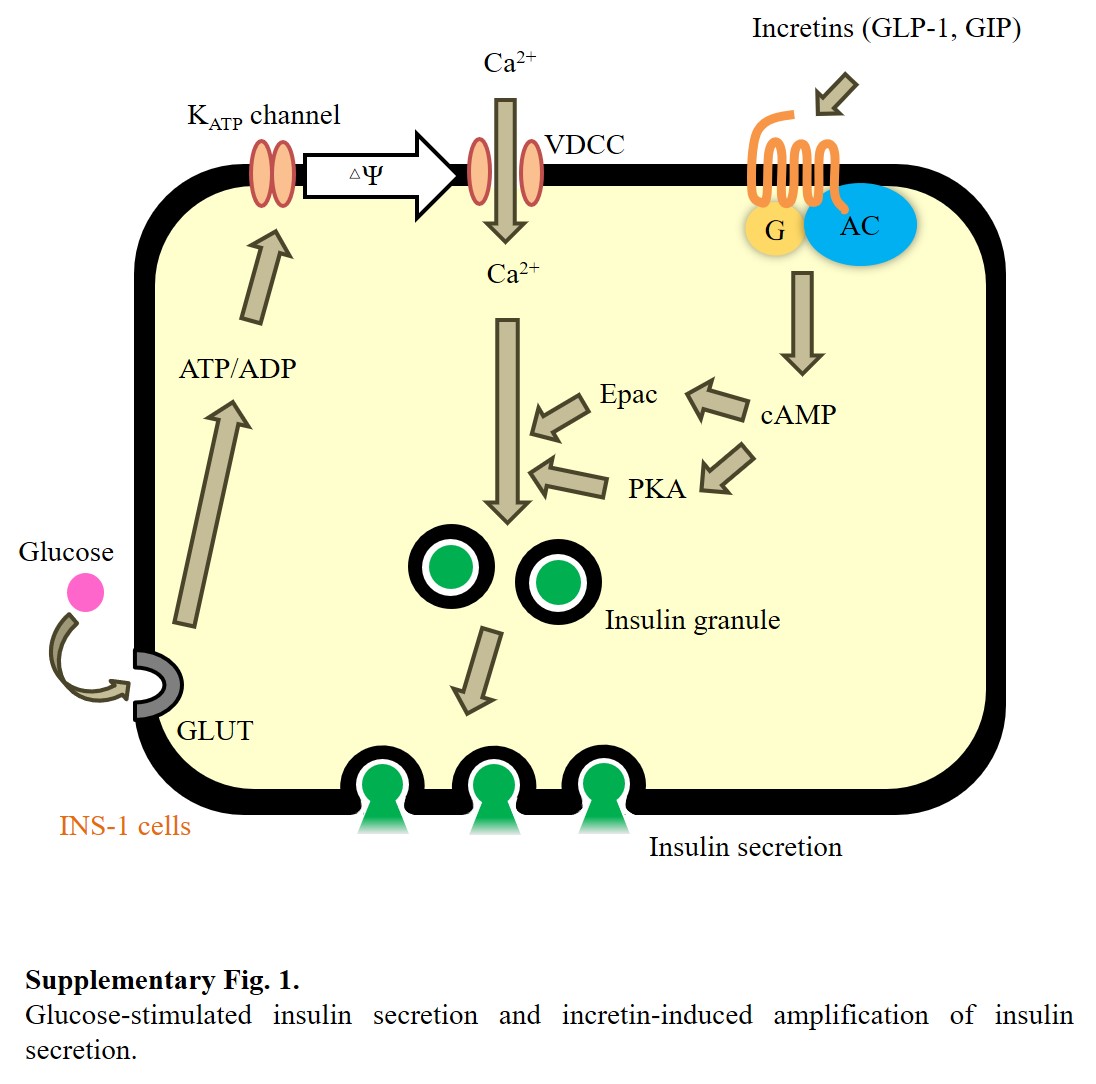

Supplement: Supplementary file 1 [file ijms-20-01995-s001.zip › supfig/SupFig1.jpg]

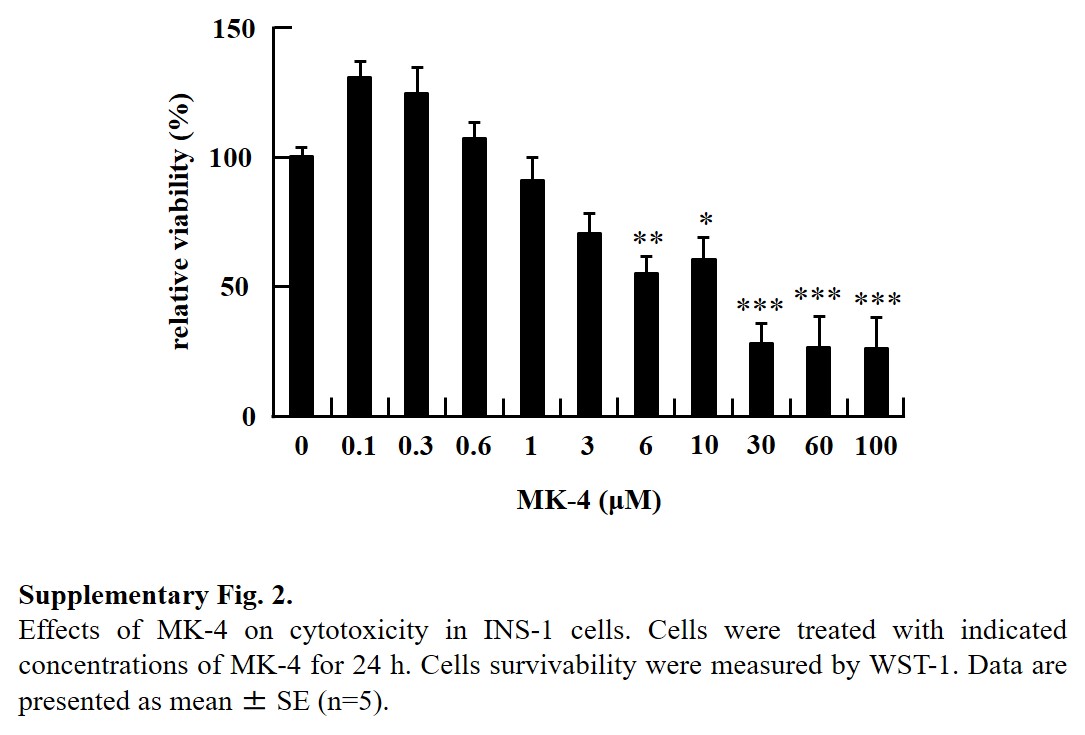

Supplement: Supplementary file 1 [file ijms-20-01995-s001.zip › supfig/SupFig2.jpg]

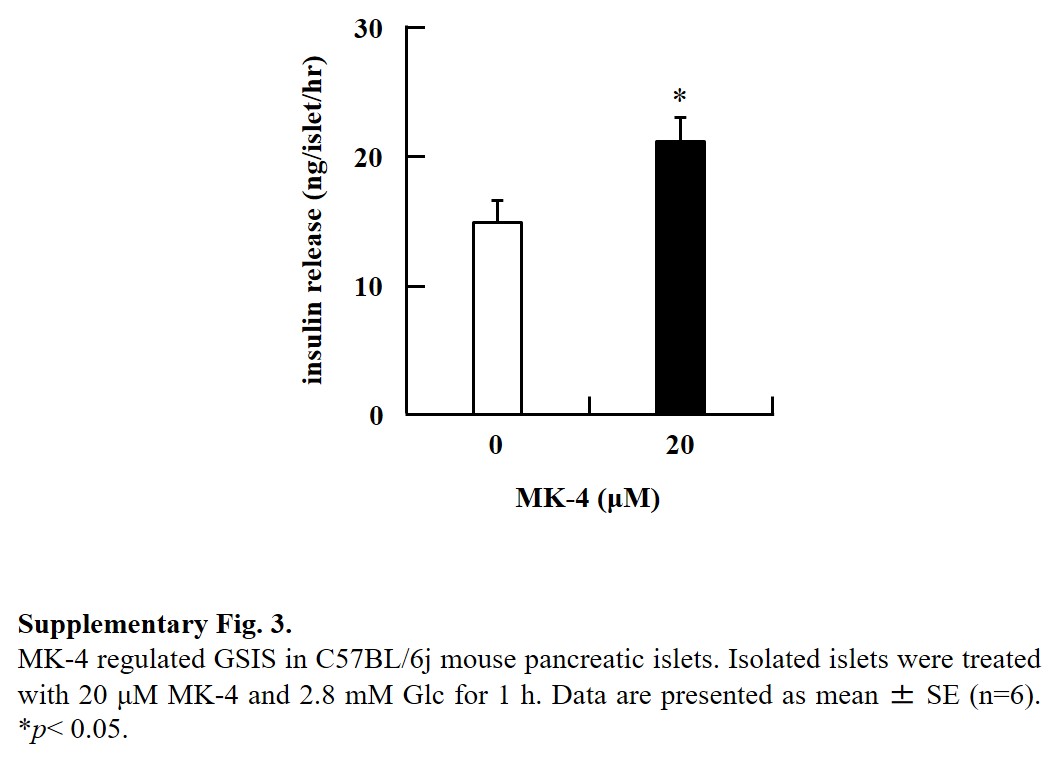

Supplement: Supplementary file 1 [file ijms-20-01995-s001.zip › supfig/SupFig3.jpg]
